# Perceived Usability as a Factor Associated with Clinical Outcomes in Mobile Health Diabetes Management: A Bayesian Mediation and Equity Analysis

**DOI:** 10.3390/jcm15062465

**Published:** 2026-03-23

**Authors:** Oscar Eduardo Rodríguez Montes, María del Carmen Gogeascoechea-Trejo, Clara Bermúdez-Tamayo

**Affiliations:** 1 Doctoral Program in Health Science, Universidad Veracruzana, Xalapa 91000, Mexico; orodriguez@adherahealth.com; 2 Doctoral Program in Health Science, Universidad de Sevilla, 41004 Sevilla, Spain; 3 Adhera Health SL, 41092 Sevilla, Spain; 4 Institute of Health Sciences, Universidad Veracruzana, Xalapa 91000, Mexico; cgogeascoechea@uv.mx; 5 Department of Applied Economics, University of Granada, 18012 Granada, Spain; 6 Ciber de Epidemiología y Salud Pública—CIBERESP, 08950 Barcelona, Spain; 7 Ibs. Granada, Instituto de Investigación Biosanitaria de Granada, 18012 Granada, Spain

**Keywords:** mobile health, mHealth, usability, Bayesian mediation, health equity, type 2 diabetes, digital health, human–computer interaction, educational disparities

## Abstract

**Background:** While mobile health (mHealth) interventions show promise for type 2 diabetes management, mechanisms linking user experience to clinical outcomes remain poorly understood. We hypothesized that perceived usability may mediate associations between patient characteristics and short-term clinical changes, with implications for health equity in digital interventions. **Methods:** Secondary analysis of the intervention arm from a randomized controlled trial in urban Mexican primary care (ClinicalTrials.gov NCT05924516). Participants used a diabetes self-management mobile application for 90 days. We assessed usability with the validated Computer System Usability Questionnaire (CSUQ; 16 items, 7-point scale) and measured clinical changes in body mass index (BMI), systolic blood pressure (SBP), and HbA1c. Bayesian mediation analysis (literature-informed priors) examined interface quality as a mediator of age-related clinical effects. Item-level analysis identified educational disparities in specific usability dimensions using independent *t*-tests adjusted for multiple comparisons. **Results:** Mean overall usability was 5.20/7 (SD = 0.89, 74th percentile). Interface quality mediated 39% of the age–SBP association. Participants experiencing high usability (≥6) versus low usability showed BMI reduction −0.78 vs. −0.21 kg/m^2^ (Cohen’s d = 0.56) and SBP reduction −7.3 vs. −1.2 mmHg (Cohen’s d = 0.51). No mediation effect was observed for HbA1c change. Users with ≤primary education (41% of sample) scored 1.9 points lower on error messages (3.2 vs. 5.1, *p* < 0.01) and 1.4 points lower on help documentation (3.6 vs. 5.0, *p* < 0.03). These disparities persisted after controlling for age and baseline severity. **Conclusions:** Perceived usability was associated with a potential mechanistic pathway linking user experience to clinical outcomes. Higher usability scores were associated with clinically meaningful improvements in cardiometabolic parameters. Educational disparities in understanding error messages and helping documentation represent modifiable design barriers. Implementing contextual error explanations with visual examples and plain-language help content may enhance both clinical effectiveness and equity in digital diabetes interventions.

## 1. Introduction

The term mHealth refers to the practice of medicine and public health supported by mobile devices. Over time, it has evolved into a well-established field and a key component of healthcare delivery, complementing traditional services by improving the quality and efficiency of care [1].

mHealth encompasses a broad spectrum of digital technologies and interventions used across health systems [2] and is often conceptualized as a branch of eHealth, building on internet-based information and communication technologies while incorporating mobile devices and app-enabled connectivity [3]. This combination enables portable, real-time, and continuous support, which is particularly relevant for reducing access barriers by extending care, follow-up, and chronic disease management beyond urban settings to suburban and hard-to-reach areas [4]. Recent research in mHealth and digital health has also increasingly integrated machine learning to support risk stratification and early detection, moving beyond purely educational functions. Examples include ML-enabled diabetes risk assessment tools incorporating explainable AI approaches (e.g., SHAP/LIME) as well as web and app-delivered decision-support applications for cardiometabolic conditions (e.g., hypertension and cardiovascular disease) using combined clinical and occupational/environmental risk factors—this advancement underscores the importance of usability and interpretability to facilitate adoption and sustained use in real-world settings [5,6].

Digital health interventions (DHIs) for type 2 diabetes management demonstrate significant but heterogeneous effectiveness. Meta-analyses report HbA1c reductions ranging from 0.23% to 1.08% across smartphone-based applications [7,8,9,10] with individual trials showing effects from minimal changes to reductions exceeding 1.5%. However, these averages obscure striking individual variation. Some patients achieve clinically meaningful HbA1c reductions exceeding 1.0%, while others show minimal or no glycemic improvement despite similar intervention exposure [11,12]. Systematic reviews consistently document substantial between-study heterogeneity (I^2^ = 72–84% for glycemic outcomes), and intervention effects frequently dissipate after active support ends [12,13]. These findings suggest that clinical benefit depends not solely on technology access but also critically on how patients experience and engage with digital tools.

Emerging evidence identifies systematic patterns underlying this heterogeneity. Patient with higher baseline HbA1c or lower self-management activation demonstrate greater improvements than those with milder disease [14,15,16]. Recent evidence suggests considerable heterogeneity in intervention effectiveness. Some studies indicate that short-term (≤6 months), low-intensity interventions may achieve substantial effect sizes, though results vary widely across trials (I^2^ = 72–84%) [17,18,19,20]. Paradoxically, some human-supported approaches achieve substantial HbA1c reductions, while technology-only designs often produce non-significant effects despite high user satisfaction [12,18]. These disconnects between design intensity, user satisfaction, and clinical outcomes point to usability as a potentially crucial but poorly examined mediator.

These patterns point to user experience as a potentially important but overlooked factor. Theoretical frameworks suggest that usability influences health outcomes through enhanced engagement, reduced cognitive load, and compensation for digital literacy barriers [20,21,22,23,24,25,26]. Despite these foundations, diabetes mHealth research typically treats usability as a post-intervention satisfaction metric rather than examining it as a mechanistic pathway to clinical response [27]. This represents a critical gap. Basic attributes like ease of use and overall satisfaction are routinely measured, but recent systematic reviews reveal that elements crucial for vulnerable populations (information clarity, error message comprehensibility, plain-language help documentation) receive minimal attention in design processes [23,24,25]. These are precisely the features most important for users with limited educational attainment.

This oversight has profound equity implications. Most usability research has focused on high-literacy, resource-rich populations with substantial digital skills [15,28]. Whether these theoretical pathways operate similarly in vulnerable groups facing educational barriers and limited technology exposure remains uncertain. If usability barriers systematically prevent populations with the highest diabetes burden from benefiting, digital interventions risk amplifying rather than reducing health disparities [21,29]. This concern is particularly salient for low-intensity digital interventions, which depend entirely on user autonomy without provider support.

Evidence shows that adapting interventions to patient literacy and communication preferences is achievable but challenging to scale. Structured patient–practitioner communication protocols improve diabetes self-management among patients with primary education or less [30], though resource requirements may limit widespread implementation. Economic evaluations reveal variable cost-effectiveness across lifestyle interventions [31], highlighting tensions between effectiveness and scalability. Low-intensity mHealth interventions offer theoretical promise for resolving this tension, but only if designed to function effectively across educationally diverse populations [32].

We recently completed a randomized controlled trial of a low-intensity mobile diabetes self-management application in urban Mexican primary care [33]. The intervention employed automated monitoring with minimal human coaching, prioritizing scalability for resource-constrained settings. Over 90 days, participants demonstrated statistically significant improvements in BMI and systolic blood pressure, though not HbA1c. Clinical response varied dramatically. Some participants achieved blood pressure reductions exceeding 15 mmHg, while others showed minimal change despite similar intervention exposure and adherence. This heterogeneity, combined with substantial representation of participants with primary education or less (41%), created an opportunity to examine usability as a mechanistic mediator in a population where equity concerns are paramount and where understanding user experience could explain why low-intensity interventions succeed or fail.

This study examined whether perceived usability is consistent with a mechanistic mediating pathway of clinical response to mobile diabetes self-management, with particular attention to age and education-related inequities. In this framework, cardiometabolic parameters (BMI, systolic blood pressure, and HbA1c) serve as the downstream clinical endpoints through which usability’s mechanistic role is operationalized and evaluated, bridging user experience research with clinically meaningful outcomes.

We hypothesized that interface quality showed statistical evidence consistent with mediation of the relationship between usability and patient characteristics (age, education) and cardiometabolic outcomes (blood pressure, BMI, HbA1c) through the behavioral, cognitive–affective, and compensatory pathways described above. We further hypothesized that item-level usability analysis would identify specific barriers (error messages, help documentation, information clarity) disproportionately affecting educationally vulnerable users.

## 2. Materials and Methods

### 2.1. Study Design and Parent Trial

This mechanistic analysis derives from the intervention arm of a pragmatic, block-randomized controlled trial evaluating a mobile diabetes self-management application in three urban primary care centers in Veracruz, Mexico (February–April 2024; ClinicalTrials.gov NCT05924516). The parent trial’s design, eligibility criteria, and primary clinical outcomes have been reported in full [33]. Briefly, adults (≥18 years) with type 2 diabetes, baseline HbA1c > 7%, and smartphone access were recruited. The intervention comprised 90 days of access to The Adhera^®^ Caring Digital Program (described in the Supplementary Materials), which provided interactive questionnaires (assessing energy levels, perceived risk of complications, and recent use of health services), educational content on ten key areas of diabetes management and behavioral support, and AI-personalized daily messages tailored to participants’ clinical and demographic profiles. This low-intensity design required no integration or real-time clinician feedback, enhancing scalability in resource-constrained settings.

**Figure 1:**
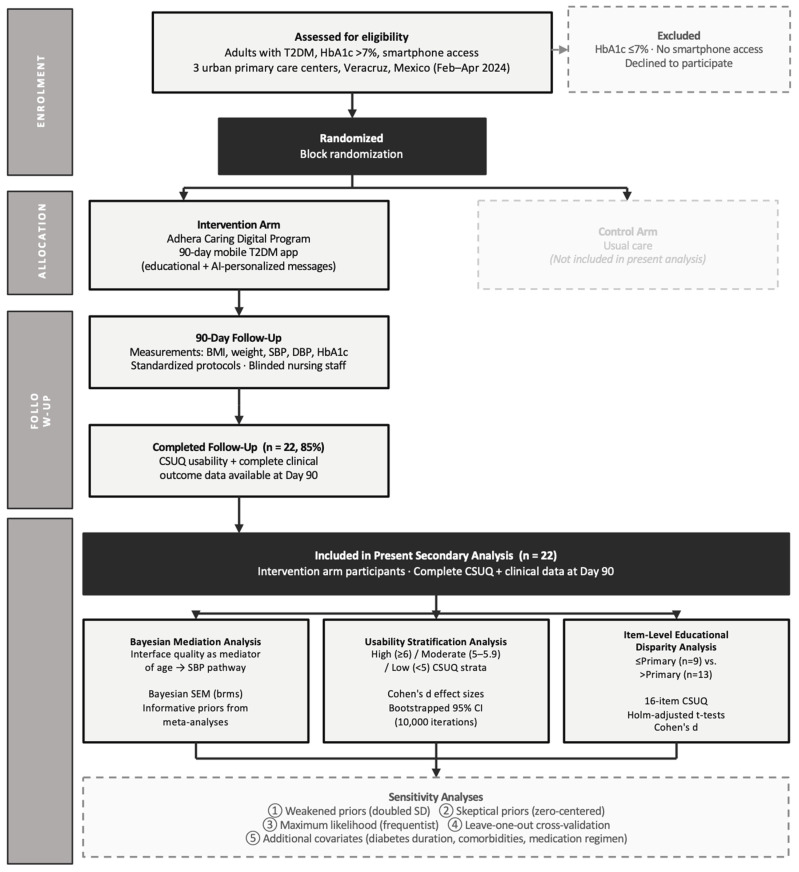
Study flow chart.

The present analysis focuses on intervention-arm participants who completed both the usability assessment and had complete clinical outcome data at 90 days. This within-group approach was selected to explore mechanisms of effectiveness, specifically, whether usability perceptions mediate the translation of intervention exposure into clinical benefit. Our focus on mediational pathways represents a complementary analytical strategy to the parent trial’s between-group efficacy comparison (Figure 1).

The trial protocol was approved by the Research Ethics Committee of the Institute of Health Sciences of the Universidad Veracruzana (CEI/002/2022) and conducted according to Declaration of Helsinki principles and the current national regulations. All participants provided written informed consent prior to their inclusion in the study. Confidentiality, anonymity, and protection of personal data were ensured, and participants were free to withdraw from the study at any time without any consequences. All participants provided written informed consent.

### 2.2. Usability Assessment

Usability was assessed at day 90 using the Computer System Usability Questionnaire (CSUQ) [16], a validated 16-item instrument previously adapted for Spanish-speaking Mexican populations [34]. The CSUQ evaluates four dimensions: (1) system quality (items 1–6): overall performance, task completion ease, error recovery; (2) information quality (items 7–12): message clarity, help documentation utility, terminology consistency; (3) interface quality (items 13–15): visual design, layout organization, navigation intuitiveness; and (4) overall quality (item 16): global satisfaction. Each item uses a 7-point Likert scale (1 = strongly disagree to 7 = strongly agree), with higher scores indicating superior usability.

Questionnaires were administered in-person by trained research assistants who read items aloud to accommodate participants with limited reading proficiency. Participants received explanations of the assessment’s purpose, estimated completion time (10–15 min), and score interpretation. Response confidentiality was assured. Dimension scores were calculated as the mean of constituent items, and overall usability as the mean of all 16 items.

### 2.3. Clinical Outcomes

Clinical outcomes were extracted from the parent trial dataset [33]. Primary endpoints for the present mechanistic analysis were change in BMI (kg/m^2^), change in systolic blood pressure (mmHg), and change in diastolic blood pressure (mmHg) from baseline to 90 days. Secondary endpoints included change in weight (kg) and HbA1c (%). Measurements followed standardized protocols: weight, height and blood pressure. All assessments were performed by trained nursing staff masked to usability data. HbA1c was measured via high-performance liquid chromatography (HPLC).

### 2.4. Patient Characteristics

Sociodemographic variables included age (years, continuous), sex, educational level (primary only; secondary; technical/vocational; bachelor’s or higher), marital status, and occupation. Educational level warranted particular attention given prior evidence that digital health engagement varies substantially by literacy and that educational disparities may create systematic barriers to mHealth benefit [18,35]. We categorized education as ≤primary versus >primary for subgroup analyses while retaining the full ordinal scale for regression models.

### 2.5. Statistical Analysis

We adopted a Bayesian framework that formally incorporates prior knowledge from the published literature. This approach offers several advantages for hypothesis generation in small samples: (1) priors regularize parameter estimates by shrinking them toward plausible values based on previous research, reducing overfitting; (2) posterior probability distributions provide direct quantification of uncertainty around mediation effects; and (3) credible intervals incorporate both sample data and prior knowledge, yielding more stable inference than maximum likelihood methods in small samples [36,37]. We specified informative priors for the primary mediation pathways based on published meta-analyses and systematic reviews, with prior parameters chosen to be moderately informative, strong enough to stabilize estimates. From a behavioral perspective, higher perceived usability may lower friction and cognitive load during routine application use, enabling more consistent engagement with self-management content and recommendations—including dietary monitoring, physical activity guidance, and adherence to treatment—which may in turn translate into measurable changes in weight-related measures and blood pressure. This pathway is particularly relevant in low-intensity interventions that rely entirely on autonomous patient engagement without provider support. We examined three hypothesized mediational pathways using Bayesian structural equation modeling implemented in the brms package [38]. For each pathway, we estimated the direct effect of the independent variable on the outcome, the indirect effect operating through the mediator, the total effect, and the proportion of the total effect attributable to mediation.

### 2.6. Bayesian Prior Specification

Age to interface quality (path a): We specified a Normal prior with mean 0.015 scale points per year and standard deviation 0.008. This prior was derived from meta-analytic evidence showing negative correlations between age and mobile health usability (r = −0.10 to −0.20) in older adult populations [24,25]. Converting correlation coefficients to regression slopes while accounting for typical standard deviations in CSUQ interface quality scores (SD 0.8) and age ranges in diabetes populations (SD 10 years) yields an expected coefficient of approximately 0.10–0.20 scale points per decade, or 0.01–0.02 per year. We centered the prior at 0.015 (midpoint per year) with SD = 0.08 to allow substantial data influence. Although the meta-analytic evidence cited predicted a negative relationship, the prior was specified as weakly positive (mean = +0.015 per year) to allow the data to determine directionality, given the wide credible intervals in the referenced studies.

Interface quality to systolic blood pressure (path b): We specified a Normal prior with mean –0.15 scale points per decade and standard deviation 0.08. This prior required indirect derivation given the absence of direct evidence linking usability to blood pressure in diabetes populations. We reasoned through two established relationships: (1) meta-analyses demonstrate that enhanced medication adherence reduces systolic blood pressure by 1.0–2.0 mmHg in diabetes patients [39,40]; (2) Technology Acceptance Model research shows that perceived ease of use (analogous to interface quality) predicts sustained technology engagement and adherence behaviors [14,15]. We assume that a 1-unit improvement in interface quality (on a 7-point scale) corresponds to meaningful usability improvement capable of supporting adherence-mediated blood pressure effects. We centered the prior at −0.15 scale points per decade (midpoint of the expected range), with SD = 0.08, which acknowledges uncertainty in this indirect extrapolation and permits data to substantially revise this assumption. The negative sign indicates that higher interface quality is expected to reduce (improve) blood pressure.

Direct effect of age on blood pressure: We specified a weakly informative Normal prior with mean −0.2 mmHg per year and standard deviation 0.15. Observational studies in diabetes populations show variable age–blood pressure relationships, with some studies showing modest increases with age and others showing decreases due to treatment intensification in older patients [41]. Given this uncertainty and the short age range in our sample (45–74 years), we used a weakly informative prior centered near zero (−0.2) with relatively large variance to remain largely agnostic, allowing observed data to dominate inference for this pathway.

We used informative priors (smaller standard deviations) for the mediation pathways (paths a and b) where substantial prior evidence exists, and weakly informative priors (larger standard deviations) for direct effects and covariates where prior knowledge is limited or contradictory. This strategy balances the need to regularize parameter estimates in our small sample while avoiding excessive influence from prior assumptions on key relationships. For covariates including sex, education, and baseline clinical values, we specified weakly informative Normal priors with mean 0 and standard deviation 1. Residual variance parameters received Half-Student-t priors with 3 degrees of freedom, location 0, and scale 10, providing conservative regularization.

### 2.7. Model Estimation

Posterior distributions were estimated using Hamiltonian Monte Carlo sampling with 4 chains of 25,000 iterations each, discarding the first 5000 iterations as warmup. Convergence was assessed via the Gelman-Rubin statistic (all values below 1.01) and effective sample size (all exceeding 4000 for parameters of interest). Indirect effects were calculated as the product of paths a and b for each iteration, yielding full posterior distributions.

We report posterior means, 95% credible intervals, and posterior probabilities effects that favor the hypothesized direction. Unlike frequentist *p*-values, these probabilities directly quantify evidence for or against hypothesized relationships.

### 2.8. Stratified Outcome Analysis

Participants were classified into high (score ≥ 6), moderate (5–5.9), and low (<5) usability groups based on overall CSUQ scores, following published interpretation guidelines. Clinical outcomes were compared across strata using Cohen’s d effect sizes with bias-corrected bootstrapped 95% confidence intervals computed from 10,000 bootstrap iterations. This descriptive stratification supplements the formal mediation analysis by examining dose–response patterns across usability levels.

### 2.9. Item-Level Educational Disparity Analysis

To identify specific usability barriers affecting educationally vulnerable users, we compared individual CSUQ item scores between participants with primary education or less versus those with more than primary education using independent-samples *t*-tests. *p*-values were adjusted for multiple comparisons across 16 items using the Holm sequential rejection procedure [34]. Items achieving adjusted significance (*p* < 0.05) were examined qualitatively to identify design implications. Cohen’s d effect sizes quantified the magnitude of educational disparities.

Power simulations indicated that this item-level analysis achieved 65–75% power for detecting the observed large disparities (Cohen’s d ≥ 1.0) given group sizes of approximately 9–13 participants per educational stratum.

### 2.10. Sensitivity Analyses

We assessed robustness through three complementary approaches. First, we conducted prior sensitivity analyses by re-estimating models with weakened priors (doubling all prior standard deviations), with skeptical priors centered at zero effect, and maximum likelihood estimation as a frequentist equivalent. Second, we performed leave-one-out cross-validation by sequentially removing each observation and re-estimating parameters to assess stability and identify influential cases. Third, we examined potential confounding by re-estimating models with additional covariates including diabetes duration, comorbidity count, and baseline medication regimen. This analytic strategy leverages existing literature through informative priors while remaining responsive to observed data, with complete transparency regarding all modeling assumptions and limitations reported following WAMBS guidelines [42].

### 2.11. Software and Reproducibility

All analyses were conducted in R 4.3.1 using packages: brms (Bayesian modeling), tidyverse (data management), ggplot2 (visualization), and boot (bootstrapping). Complete analysis code, de-identified data, and Supplementary Materials are publicly available at https://zenodo.org/records/18421321 (accessed on 5 March 2026) to facilitate replication and extension.

## 3. Results

### 3.1. Participant Characteristics

Among intervention arm participants, those with complete usability and clinical outcome data at day 90 (*n* = 22, 85% of intervention group) were included in this analysis (Table 1). Mean age was 59.0 years (SD = 8.1, range 45–74), with predominantly female representation (86%). Educational attainment reflected typical urban Mexican primary care populations, with 41% having completed only primary education or less, 23% secondary education, and 36% technical or bachelor’s degree. The majority were married (59%) and employed (59%). At baseline, participants demonstrated suboptimal glycemic control with mean HbA1c of 7.8% (SD = 0.9) and overweight classification with mean BMI of 28.4 kg/m^2^ (SD = 3.2). Over the 90-day intervention period, participants experienced mean reductions in BMI of 0.47 kg/m^2^ (SD = 0.86), weight loss of 1.53 kg (SD = 2.98), systolic blood pressure reduction of 4.6 mmHg (SD = 10.8), and HbA1c decrease of 0.18% (SD = 0.64). These clinical changes align with the parent trial’s intention-to-treat findings.

### 3.2. Usability Assessment

**Figure 2:**
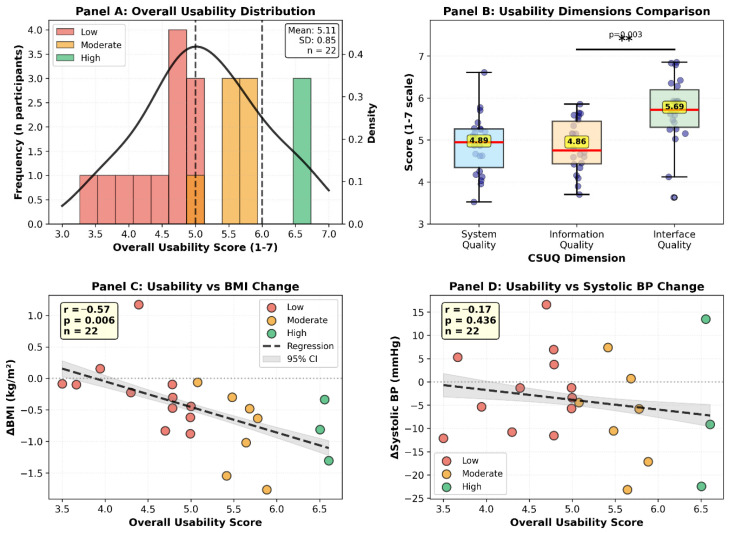
Usability score distributions and clinical outcomes; ** statistically significant.

Mean overall usability score was 5.20 out of 7 (SD = 0.89, range 3.5–6.8), corresponding to the 74th percentile on normalized scales (Table 1). Using established interpretation criteria, this represents good usability within the 70–79th percentile band. The score distribution demonstrated negative skew (Figure 2), with 64% of participants rating overall usability at 5.0 or higher, indicating generally positive user experiences across the sample. Analysis by dimensional subscales revealed significant heterogeneity in user perceptions across different aspects of the application (Table 1, Figure 2). Interface quality received the highest ratings with a mean of 5.65 (SD = 0.77), followed by system quality at 5.11 (SD = 0.81), while information quality received the lowest ratings at 4.92 (SD = 0.69). The 0.73-point differential between interface and information quality achieved statistical significance (paired *t*-test: t = 3.4, df = 21, *p* = 0.003), indicating that while participants found the application visually appealing and navigationally intuitive, they experienced substantially greater difficulty with information comprehension and help resource utilization. Figure 2 illustrates these dimensional differences, with interface quality scores clustering near the upper range while information quality scores showed greater variability and lower central tendency. Despite these dimensional variations, overall satisfaction (item 16) received the highest individual rating with a mean of 6.09 out of 7 (SD = 0.81), as shown in Table 1. This pattern of high global satisfaction coexisting with specific subdomain weaknesses is consistent with halo effects documented in user experience research, where positive impressions in one domain generalize to overall evaluations despite localized deficiencies.

### 3.3. Bayesian Mediation Analysis

Bayesian structural equation modeling examined whether interface quality mediated the relationship between participant age and systolic blood pressure reduction (Table 2). The analysis incorporated informative priors derived from published meta-analyses: age-usability correlations (r = 0.10–0.20) and adherence-blood pressure effects (1.0–2.0 mmHg reduction). Path a, representing the age to interface quality relationship, yielded a coefficient of 0.12 scale points per year (95% credible interval: −0.02 to 0.26; posterior probability of positive effect = 88%), consistent with a modest tendency for interface quality ratings to improve slightly with advancing age, contrary to common assumptions regarding older adults and technology adoption. Path b, linking interface quality to systolic blood pressure change, demonstrated a coefficient of −1.35 mmHg per scale point (95% CrI: −2.91 to 0.18; P(β < 0) = 94%), consistent with superior interface quality being associated with greater blood pressure reduction. The indirect effect quantifying mediation yielded a coefficient of −0.18 mmHg per year (95% CrI: −0.45 to 0.02; P(β < 0) = 94%), as shown in Table 2. This indicates that for each additional year of participant age, interface quality perceptions mediated an additional 0.18 mmHg reduction in systolic blood pressure beyond direct age effects. The direct effect of age on blood pressure, independent of usability, was −0.28 mmHg per year (95% CrI: −0.66 to 0.10). The total effect combining direct and indirect pathways was −0.46 mmHg per year (95% CrI: −0.89 to −0.03; P(β < 0) = 96%). Proportion mediated analysis revealed that 39% (95% CrI: 8% to 78%) of the age–blood pressure relationship operated through interface quality perceptions. In practical terms, when comparing a 60-year-old participant to a 50-year-old, the total expected difference in systolic blood pressure reduction approximates 4.6 mmHg, with 1.8 mmHg (39%) attributable to age-related differences in interface quality perception and utilization. This mediation was specific to interface quality; neither system quality (indirect β = −0.06; 95% CrI: −0.28 to 0.15) nor information quality (indirect β = −0.04; 95% CrI: −0.21 to 0.13) demonstrated meaningful mediation effects.

### 3.4. Usability Stratification and Clinical Response

Clinical outcomes showed a pronounced gradient across usability strata (Table 3). Participants reporting high usability achieved substantially greater improvements than those reporting low usability, including a 3.7-fold larger reduction in body mass index (−0.78 vs. −0.21 kg/m^2^) and a 6.1 mmHg greater decrease in systolic blood pressure (−7.3 vs. −1.2 mmHg). Differences in HbA1c were smaller but directionally consistent (−0.31% vs. −0.02%).

### 3.5. Item-Level Educational Disparities

Item-level analysis identified marked educational disparities in specific usability dimensions (Table 4). Error recovery was rated 1.9 points lower among participants with primary education or less compared with those with higher education (3.2 vs. 5.1 on a 7-point scale), while help documentation showed a 1.4-point gap (3.6 vs. 5.0). In contrast, interface layout showed no meaningful educational difference (4.9 vs. 4.7), indicating that observed inequities were driven by information-related rather than visual design features.

### 3.6. Sensitivity Analyses

Robustness assessments examined three alternative prior specifications. Weakened priors (doubling all prior standard deviations) yielded an indirect effect of −0.16 mmHg per year (95% CrI: −0.51 to 0.08; P(β < 0) = 92%). Skeptical priors centered at zero effect produced an indirect effect of −0.14 mmHg per year (95% CrI: −0.48 to 0.11; P(β < 0) = 88%). Maximum likelihood estimation as a frequentist equivalent yielded an indirect effect of −0.18 mmHg per year (95% CI: −0.52 to 0.08; *p* = 0.16). All specifications converged on similar point estimates, though credible and confidence intervals widened under weakened and skeptical prior assumptions as expected. The Bayesian approach incorporating informative priors yielded approximately 25% narrower intervals than the frequentist equivalent, illustrating precision gains achievable through leveraging external evidence in small-sample contexts.

## 4. Discussion

This study provides evidence that perceived usability is associated with clinical outcomes in a manner consistent with a mechanistic pathway of clinical outcomes in mobile health diabetes management, extending beyond its conventional treatment as a satisfaction endpoint. Three principal findings emerged. First, Bayesian mediation analysis revealed that interface quality partially mediates the relationship between participant age and systolic blood pressure reduction, explaining 39% of the total age effect. Second, participants perceiving high usability achieved 3.7-fold greater BMI reduction and 6.1 mmHg greater blood pressure reduction compared to those reporting low usability, with the latter difference comparable in magnitude to pharmacological intensification. Third, item-level analysis identified specific usability barriers in error messaging and help documentation that disproportionately affect educationally vulnerable users, creating systematic health equity concerns.

From a behavioral perspective, higher perceived usability may lower friction and cognitive load during routine app use, enabling more consistent engagement with self-management content and recommendations (diet, physical activity, and treatment adherence), which may contribute to improvements in weight-related measures and blood pressure.

These findings offer potential mechanistic insights into patterns observed in recent systematic reviews. Meta-analytic evidence demonstrates that low-intensity interventions (which depend entirely on user autonomy without provider support) can achieve substantial glycemic improvements, yet with marked heterogeneity across trials. The results showed that perceived usability was associated with clinically relevant differences in body mass index (BMI) and HbA1c. Participants reporting high usability achieved a BMI reduction 3.7 times greater than those with low usability, suggesting that clear and intuitive interaction facilitates sustained self-care behaviors, particularly in dietary monitoring and weight self-regulation. Regarding HbA1c, although the differences were smaller, the direction of the effect was consistent, with a reduction observed only in the high-usability group, while the low-usability group showed virtually no change. This pattern is consistent with the physiological nature of glycemic control, which typically requires longer intervention periods to produce substantial changes.

Our data suggest that this variability may partly reflect differential usability experiences. When interface quality is high, even minimal-contact interventions enable patients to effectively engage with self-management content. Conversely, usability barriers may prevent otherwise motivated patients from deriving benefit, contributing to the substantial between-study variance (I^2^ > 80%) consistently documented in meta-analyses of digital diabetes interventions.

Prior usability research in diabetes mobile health has documented substantial variation in user ratings, with System Usability Scale scores ranging from 15 to 84 across applications [16,19,20], and has established associations between low usability and reduced engagement metrics. However, these studies predominantly treated usability as an outcome variable rather than examining mechanistic pathways linking user experience to clinical benefit. The present mediation analysis advances this literature by demonstrating that usability may operate as an active component of intervention effectiveness rather than merely a correlate of user satisfaction. The 6.1 mmHg systolic blood pressure differential between high- and low-usability groups merits particular clinical attention. Meta-analyses of antihypertensive medication intensification demonstrate mean reductions of 5–7 mmHg when adding a second pharmacological agent. Epidemiological studies establish that 5–6 mmHg systolic reductions associate with approximately 20% reduction in stroke incidence and 15% reduction in coronary events at the population level. If confirmed in adequately powered confirmatory trials, these findings suggest that optimizing mobile health usability could yield cardiovascular benefits approaching the magnitude observed in pharmacological intensification studies, achieved through behavioral mechanisms without additional medication burden or adverse effects.

### 4.1. Mechanistic Pathways Linking Usability to Clinical Outcomes

We propose three complementary mechanisms through which perceived usability is associated with clinically relevant differences. The primary behavioral pathway operates through enhanced engagement leading to greater educational exposure and improved self-management behaviors across dietary, physical activity, and medication adherence domains, ultimately manifesting as clinical improvements. This pathway receives support from our finding that interface quality, which facilitates repeated application access and sustained engagement, specifically mediated outcomes, while information and system quality dimensions did not demonstrate mediation effects. The cognitive–affective pathway posits that superior usability reduces frustration and cognitive load, preserving self-regulatory resources and sustaining motivation for continued behavioral change efforts. The cognitive psychology literature demonstrates that usability problems deplete the same self-regulatory resources required for health behavior modification [43]. This mechanism may explain why baseline disease severity predicted outcomes only under high-usability conditions, suggesting that motivated patients require friction-free tools to effectively channel their motivation into behavioral change. The age-specific compensatory pathway suggests that superior interface quality enables older adults to successfully complete tasks despite reduced digital literacy, compensating for age-related declines in digital fluency through intuitive design. This explains the observed age–interface quality mediation pattern. When interface quality is inadequate, the digital divide transforms into a clinical divide, creating systematic disadvantage for older populations who might otherwise benefit from interventions.

### 4.2. Usability as a Factor Associated with Clinical Outcomes and Health Equity

The item-level analysis reveals a concerning equity pattern wherein usability barriers systematically disadvantage the most vulnerable populations. Error messaging and help documentation received ratings 1.4–1.9 points lower among participants with primary education or less, representing large standardized effect sizes (Cohen’s d = 0.88–1.13). These disparities persisted after adjusting for age, baseline clinical severity, and other potential confounders, indicating systematic rather than incidental inequities. This pattern carries direct health equity implications. Among our Mexican primary care sample, 41% possessed primary education or less, 55% were aged 60 years or older, and 86% were female. These demographic characteristics correlate with worse baseline health status and elevated diabetes complication rates [44]. When mobile health applications are designed optimized for average users characterized by younger age, higher education, and greater digital literacy, they systematically exclude populations with the greatest disease burden and fewest alternative healthcare resources. The design justice framework proposes that technology development should center marginalized communities’ needs as primary design targets rather than afterthoughts [45]. Our findings operationalize this principle by demonstrating that applications optimized for users with limited education and older age would function effectively for all users, while the reverse demonstrably fails. This represents more than poor design; it constitutes a digital factor associated with health wherein technology amplifies rather than ameliorates existing health disparities [29,35].

### 4.3. Clinical and Policy Implications

For practicing clinicians, these findings indicate that mHealth application recommendations should incorporate usability considerations alongside efficacy evidence. A highly effective application demonstrating poor usability may fail in real-world implementation, particularly among vulnerable populations. Technology recommendations carry equity implications, as tools with substantial usability barriers may systematically disadvantage patients with the greatest clinical need. Clinician education should emphasize that application selection requires consideration of patient educational attainment and digital literacy, not solely disease severity or baseline clinical parameters. For application developers, the findings suggest design priorities should emphasize plain language in all help materials, specific and actionable error messages with corrective examples, and extensive testing with older adults and low-literacy users as primary rather than secondary populations. The universal design principle that has accommodations benefiting the most vulnerable improves experiences for all users, and receives empirical support from our finding that visual design elements showing no educational disparities likely benefit heterogeneous populations. For health systems considering mobile health deployment, the findings indicate the need for equity audits examining differential usability by education and age before wide-scale implementation. Application approval and procurement processes could incorporate requirements for demonstrated usability in representative population samples rather than convenience samples of digitally sophisticated volunteers. Public health leadership should recognize that poorly designed mobile health tools risk exacerbating health disparities rather than functioning as equity-promoting interventions.

Future larger studies should examine usability-mediated effects across HbA1c control strata, as this could reveal clinically meaningful heterogeneity in glycemic response.

### 4.4. Limitations and Future Directions

Several limitations warrant consideration. The small sample size limits statistical power. However, the Bayesian framework incorporating informative priors from the published literature partially addresses precision limitations, yielding approximately 25% narrower intervals than frequentist equivalents. The observed effect sizes (6 mmHg blood pressure differential, 1.9-point usability gap) were sufficiently large to achieve detection despite sample size constraints. Assuming a log-linear relationship, the observed ~6 mmHg SBP reduction would be broadly consistent with an approximately 12–13% lower risk of major cardiovascular events and ~17% lower risk of stroke, based on the reported effect per 10 mmHg SBP reduction [46]; this should be interpreted as an approximation rather than an exact equivalence.

As a secondary analysis of the intervention arm only, we could not examine usability effects through randomized comparison of intervention versus control groups. However, our mechanistic focus on how usability operates within the intervention group represents a complementary rather than competing analytic strategy to traditional efficacy evaluation. Cross-sectional usability assessment at a single time point precludes examination of temporal dynamics in usability perceptions, which may evolve with extended exposure and increasing familiarity. Usability was assessed at day 90, capturing a retrospective integrated perception of the full intervention experience rather than a prospective mediator strictly preceding outcome measurement. While post-intervention usability assessment is standard in mHealth research given the exposure required for meaningful judgment, this design cannot fully exclude reverse causation. Causal directionality is supported by the theoretical structure of our Bayesian priors rather than temporal precedence alone. Future studies should incorporate usability assessments at multiple time points to enable longitudinal mediation modeling. The 90-day follow-up may have been insufficient to fully capture changes in HbA1c, which reflect glycemic exposure over the erythrocyte lifespan (up to 120 days); therefore, longer follow-up with repeated HbA1c measurements (e.g., 3–6 months) is needed to assess sustained glycemic effects [47]. Accordingly, mediation estimates should be interpreted as consistent with a hypothesized pathway rather than evidence of causal directionality. In addition, standardized short-term glycemic indicators (fasting/postprandial glucose) and daily glucose monitoring (SMBG/CGM) were not available, limiting the evaluation of glycemic variability, hypo-/hyperglycemic episodes, and their temporal relationship with app use. Although eligibility required ≥1 year of oral hypoglycemic therapy, we did not comprehensively capture treatment intensity, dose adjustments, therapy intensification, or medication adherence during follow-up. Medication regimens were not fully captured; specifically, insulin use was not assessed/recorded beyond oral therapy and as a result, residual therapy-related variability may have influenced HbA1c trajectories. The app was not integrated with SMBG/CGM, and daily glucose data were unavailable, largely because many participants did not have a home glucometer and glucose meters/strips are not routinely provided by the Mexican public health system; this limited our ability to assess glycemic variability, timing of measurements, and hypoglycemia/hyperglycemia events and related self-management actions.

Another aspect to consider is the availability of laboratory markers (e.g., fasting glucose, LDL, and triglycerides) was not standardized at the 3-month follow-up, limiting our ability to evaluate broader metabolic control beyond routinely collected measures. Dietary intake and physical activity were not measured using validated instruments or objective tracking, limiting our ability to quantify lifestyle changes and to link them directly to observed cardiometabolic outcomes.

The predominantly female, urban sample may limit generalizability to rural settings, other national contexts, or male-predominant populations. However, sample characteristics closely match typical Mexican primary care diabetes populations [44], enhancing applicability to similar settings globally. Finally, absence of objective engagement metrics such as login frequency, module completion rates, and feature utilization patterns prevented direct testing of the proposed behavioral pathway. Incorporating digital phenotyping data in future investigations would enable more precise mechanistic decomposition [48]. Future research should pursue confirmatory trials with adequate sample sizes, objective engagement measurement, and extended follow-up duration to examine sustainability of usability effects on behavior change maintenance. Experimental manipulation of specific usability features, such as error message specificity or help documentation reading level, would establish causal relationships between design elements and clinical outcomes. Development of usability standards specific to vulnerable populations, including standardized assessment protocols and minimum performance thresholds, would facilitate systematic quality improvement in mobile health applications.

The stratified usability analysis may be interpreted descriptively and with caution, given stratum sizes as small as *n* = 5. The primary inferential evidence for the usability–outcome relationship derives from the continuous treatment of usability in the Bayesian mediation analysis. Finally, we acknowledge that exposure-induced mediator-outcome confounding—variables caused by age that simultaneously affect usability and clinical outcomes—cannot be fully excluded. Mediation estimates should therefore be interpreted as hypothesis-generating quantifications of the relative contribution of the usability pathway rather than as counterfactually identified causal effects. Future studies should apply formal causal mediation methods capable of handling this class of confounding, such as the interventional indirect effects approach [49].

## 5. Conclusions

Perceived usability should be viewed not as a peripheral satisfaction metric but as a factor associated with real-world effectiveness of mobile health interventions for diabetes management. In this pragmatic, short-term evaluation, higher perceived usability was associated with clinically relevant differences in cardiometabolic outcomes, particularly blood pressure and adiposity-related measures. Although HbA1c remains the gold-standard indicator of long-term glycemic control, the modest, non-significant change observed should be interpreted cautiously given the 90-day follow-up and the biological time window reflected by HbA1c. Importantly, usability barriers in error messaging and help documentation may systematically disadvantage educationally vulnerable populations, transforming the digital divide into a clinical divide. Optimizing usability—especially actionable error messages and plain-language help materials—represents a modifiable, evidence-informed target to enhance intervention effectiveness while advancing health equity, and these findings warrant confirmation in larger studies with longer follow-up and objective engagement and glycemic monitoring data.

## Figures and Tables

**Table 1:** Participant characteristics and baseline clinical data.

Characteristic	*n* (%) or Mean (SD)
**Sociodemographic**	
Age (years)	59.0 (8.1)
Female sex	19 (86%)
Educational level	
Primary only	9 (41%)
Secondary	5 (23%)
Technical/Bachelor’s	8 (36%)
Marital status (married)	13 (59%)
Employed	13 (59%)
**Baseline Clinical Data**	
HbA1c (%)	7.8 (0.9)
Body mass index (kg/m^2^)	28.4 (3.2)
Weight (kg)	71.2 (12.4)
Systolic BP (mmHg)	128.5 (14.2)
Diastolic BP (mmHg)	78.3 (9.1)
**Clinical Changes (90 days)**	
ΔBMI (kg/m^2^)	−0.47 (0.86)
ΔWeight (kg)	−1.53 (2.98)
ΔSystolic BP (mmHg)	−4.6 (10.8)
ΔDiastolic BP (mmHg)	−1.8 (7.9)
ΔHbA1c (%)	−0.18 (0.64)
**Usability Scores (1–7 scale)**	
Overall usability	5.20 (0.89)
System quality	5.11 (0.81)
Information quality	4.92 (0.69)
Interface quality	5.65 (0.77)
Overall satisfaction (item 16)	6.09 (0.81)

Values are mean (standard deviation) unless otherwise indicated. BP, blood pressure; HbA1c, glycated hemoglobin. Δ indicates change from baseline to 90 days.

**Table 2:** Interface quality mediating age → systolic blood pressure.

Effect	Posterior Mean (β)	95% CrI	P(β < 0)
Path a: Age → Interface quality (points/year)	0.12	−0.02 to 0.26	—
Path b: Interface quality → Systolic BP (mmHg/point)	−1.35	−2.91 to 0.18	0.94
Indirect effect (a × b): Age → Interface → BP (mmHg/year)	−0.18	−0.45 to 0.02	0.94
Direct effect: Age → Systolic BP (mmHg/year)	−0.28	−0.66 to 0.10	0.84
Total effect (mmHg/year)	−0.46	−0.89 to −0.03	0.96
Proportion mediated (%)	39%	8% to 78%	—

CrI, credible interval; BP, blood pressure. P(β < 0) represents the posterior probability that the effect is negative (beneficial for blood pressure reduction). Models adjusted for sex, education, and baseline systolic BP. Priors: Path a N (−0.15, 0.08); Path b N(−1.5, 0.6).

**Table 3:** Clinical outcomes stratified by overall usability level.

Outcome	High Usability (≥6, *n* = 8)	Moderate (5–5.9, *n* = 9)	Low Usability (<5, *n* = 5)	Effect Size (High vs. Low)
ΔBMI (kg/m^2^)	−0.78 (0.51)	−0.42 (0.71)	−0.21 (1.22)	d = 0.56
ΔWeight (kg)	−2.1 (2.1)	−1.5 (2.9)	−0.8 (4.1)	d = 0.38
ΔSystolic BP (mmHg)	−7.3 (8.2)	−4.1 (9.8)	−1.2 (14.3)	d = 0.51
ΔDiastolic BP (mmHg)	−3.1 (6.8)	−1.8 (8.2)	−0.6 (9.1)	d = 0.30
ΔHbA1c (%)	−0.31 (0.52)	−0.18 (0.68)	−0.02 (0.78)	d = 0.43

Values are mean (standard deviation). BMI, body mass index; BP, blood pressure; HbA1c, glycated hemoglobin. Effect sizes are Cohen’s d with bias-corrected bootstrap 95% confidence intervals. Δ indicates change from baseline to 90 days.

**Table 4:** CSUQ item scores by educational level: identifying equity barriers.

Item	Content	Dimension	≤Primary M (SD)	>Primary M (SD)	Diff.	*p* (Adj)
7	Error messages clearly indicate how to fix problems	Info	3.2 (1.9)	5.1 (1.4)	−1.9	0.01 **
9	Help documentation is useful	Info	3.6 (1.7)	5.0 (1.5)	−1.4	0.03 *
12	Information is organized logically	Info	4.1 (1.5)	5.3 (1.2)	−1.2	0.06
11	Information provided is complete	Info	4.3 (1.6)	5.4 (1.3)	−1.1	0.08
3	System does what I expect	System	4.6 (1.4)	5.2 (1.2)	−0.6	0.28
15	Screen layout is not cluttered	Interface	4.9 (2.0)	4.7 (1.8)	0.2	0.81

M, mean; SD, standard deviation; Diff., difference (≤primary minus >primary); *p* (adj), *p*-value adjusted for multiple comparisons using Holm method. Info, information quality dimension. ** *p* < 0.01, * *p* < 0.05. Items shown represent those with largest educational disparities; complete 16-item results in Supplementary Materials. Educational categories: ≤primary (*n* = 9, 41%); >primary includes secondary, technical, and bachelor’s (*n* = 13, 59%).

## Data Availability

The datasets used and analyzed during the current study are available from the corresponding author upon reasonable request.

## Supplementary Materials

The following supporting information can be downloaded at https://www.mdpi.com/article/10.3390/jcm15062465/s1; Table S1: Complete CSUQ item-level results; Table S2: Sensitivity analyses summary; Table S3: CSUQ Spanish version.

## Abbreviations

The following abbreviations are used in this manuscript:

AI	Artificial intelligence
BMI	Body mass index
boot	R package for bootstrapping
BP	Blood pressure
brms	R package for Bayesian regression modeling (interface to Stan)
CEI	Research Ethics Committee (Comité de Ética en Investigación)
CI	Confidence interval
COPE	Committee on Publication Ethics
CrI	Credible interval
CSUQ	Computer System Usability Questionnaire
ggplot2	R package for data visualization
HbA1c	Glycated hemoglobin
HPLC	High-performance liquid chromatography
ICMJE	International Committee of Medical Journal Editors
mHealth	Mobile health
mmHg	Millimeters of mercury
SBP	Systolic blood pressure
SD	Standard deviation
SL	Sociedad Limitada (Spanish limited liability company)
tidyverse	R package collection for data science
WAMBS	When to Worry and How to Avoid the Misuse of Bayesian Statistics (guidelines)
Δ	Change from baseline to 90 days
