# Supplementary material for: Perceived Usability as a Factor Associated with Clinical Outcomes in Mobile Health Diabetes Management: A Bayesian Mediation and Equity Analysis
Source: J Clin Med. 2026 Mar 23;15(6):2465. doi: 10.3390/jcm15062465 (PMC13026862; doi:10.3390/jcm15062465)
Supplement: Supplementary file 1 [file jcm-15-02465-s001.zip › jcm-4174179-supplementary.pdf]

SUPPLEMENTARY MATERIALS

Perceived Usability as a Factor Associated with Clinical Outcomes in Mobile Health Diabetes Management: A Bayesian Mediation and Equity Analysis

Oscar Eduardo Rodríguez-Montes, MD, MSc [1,2,3], María del Carmen Gogeascoechea-Trejo, PhD[4], Clara Bermúdez-Tamayo, PhD[5,6,7*]

TABLE OF CONTENTS

Description of the Mobile Application Intervention: Adhera Caring Digital Program ®

Supplementary Table S1. Complete CSUQ item-level results

Supplementary Table S2. Sensitivity analyses summary

Supplementary Table S3. CSUQ Spanish version

Description of the Mobile Application Intervention: Adhera Caring Digital Program ®

Participants assigned to the intervention group received a temporary license code to access the *Adhera Caring Digital Program®* for T2DM, a mobile health application with a developed to support diabetes self-management. Upon downloading and installing the app, participants completed a registration process in which they provided demographic and clinical information, including age, sex, height, weight, history of diabetes-related complications, and personal interests related to disease management.

Following registration, the app displayed a welcome message outlining the objectives of the program and explaining its main functionalities. The message also introduced the structure and components of the application, which was designed to offer personalized recommendations to prevent complications associated with type 2 diabetes mellitus.

The app featured a simple, user-friendly interface, allowing customization of notifications and display preferences. Users could personalize their experience by modifying the visual layout and choosing the a profile photo, and other preferences at the menu display. The application was structured around three main functional modules:

1. **Interactive Questionnaires**: These included assessments of energy levels, perceived risk of complications, and recent use of health services. Questionnaires were administered with variable frequency (weekly, biweekly, or monthly), depending on user interaction and profile and objective.
2. **Educational and Behavioral Support Content**:The app provided access to structured educational materials covering ten key areas of diabetes management. These included nutrition, physical activity, medication adherence, glucose monitoring, problem-solving, risk reduction, healthy coping, fatigue reduction, sleep quality, and complication prevention. The content was complemented by challenges in the form of short quizzes to reinforce knowledge retention and user engagement.
3. **Personalized Messaging System**:Daily messages were delivered using a recommender system supported by artificial intelligence algorithms. These messages were tailored based on “metafeatures” derived from each participant’s demographic and clinical profile. Users could rate the relevance and helpfulness of each message on a five-point scale, allowing the system to refine future recommendations.

Throughout the 90-day intervention, educational content and challenges were continuously accessible, while AI-generated messages were delivered once per day. The dynamic questionnaire module adapted its frequency of interaction to optimize user engagement and data collection.

SUPPLEMENTARY TABLE S1

Complete CSUQ Item-Level Results for All 16 Items

#	Item Content	Overall Mean (SD)	≤Primary (n=9)	>Primary (n=13)	Diff	p-adj	Cohen's d
1	Overall, I am satisfied with how easy it is to use this system	5.8 (0.9)	5.6 (1.0)	6.0 (0.8)	-0.4	0.34	0.44
2	It was simple to use this system	5.5 (1.1)	5.2 (1.3)	5.7 (0.9)	-0.5	0.29	0.44
3	I could effectively complete the tasks using this system	5.0 (1.3)	4.6 (1.4)	5.2 (1.2)	-0.6	0.28	0.47
4	I was able to complete the tasks quickly	5.1 (1.0)	4.8 (1.2)	5.3 (0.9)	-0.5	0.31	0.47
5	I was able to efficiently complete tasks	5.2 (0.9)	4.9 (1.1)	5.4 (0.8)	-0.5	0.26	0.51
6	I felt comfortable using this system	5.6 (1.0)	5.4 (1.2)	5.7 (0.9)	-0.3	0.48	0.28
**7**	**Whenever I made a mistake, I could recover easily and quickly**	**4.1 (1.8)**	**3.2 (1.9)**	**5.1 (1.4)**	**-1.9**	**0.01****	**1.13**
8	The information provided was clear	5.0 (1.2)	4.5 (1.4)	5.3 (1.0)	-0.8	0.14	0.64
**9**	**It was easy to find the information I needed**	**4.3 (1.6)**	**3.6 (1.7)**	**5.0 (1.5)**	**-1.4**	**0.03***	**0.88**
10	The information was effective in helping me complete tasks	4.9 (1.3)	4.4 (1.5)	5.2 (1.1)	-0.8	0.15	0.60
11	*The organization of information was clear*	*4.8 (1.5)*	*4.3 (1.6)*	*5.4 (1.3)*	*-1.1*	*0.08†*	*0.76*
12	*The interface gave clear messages*	*4.7 (1.4)*	*4.1 (1.5)*	*5.3 (1.2)*	*-1.2*	*0.06†*	*0.91*
13	The interface was pleasant	5.8 (0.8)	5.7 (0.8)	5.9 (0.7)	-0.2	0.52	0.27
14	I liked using the interface	5.8 (0.7)	5.7 (0.8)	5.9 (0.7)	-0.2	0.52	0.27

15	Overall, the system layout was cluttered (reverse)	4.8 (2.0)	4.9 (2.0)	4.7 (1.8)	0.2	0.81	0.11
16	Overall, I am satisfied with this system	6.1 (0.8)	6.0 (0.9)	6.2 (0.7)	-0.2	0.54	0.25

*All values are mean (SD) on 1-7 scale (higher = better usability, except item 15 which is reverse-scored). P-values adjusted for 16 comparisons using Holm method. **p<0.01; *p<0.05; †p<0.10 (borderline significance). Bold = statistically significant; Italics = borderline significant. Educational groups: ≤Primary = completed primary school or less (n=9); >Primary = secondary education or higher (n=13).*

SUPPLEMENTARY TABLE S2

Sensitivity Analyses for Primary Mediation Model

Analysis	Indirect Effect β	95% CI/CrI	P-value / P(β<0)
**Primary analysis (Bayesian, informative priors)**	**-0.18**	**-0.45 to 0.02**	**94%**
Weakened priors (SD × 2)	-0.16	-0.51 to 0.08	92%
Skeptical priors (centered at 0)	-0.14	-0.48 to 0.11	88%
Flat/vague priors (SD × 10)	-0.17	-0.57 to 0.13	87%
Frequentist (maximum likelihood)	-0.18	-0.52 to 0.08	p=0.16
Adjusted for diabetes duration	-0.17	-0.44 to 0.04	93%
Adjusted for comorbidity count	-0.16	-0.44 to 0.03	92%
Adjusted for baseline medications	-0.16	-0.42 to 0.02	93%
Fully adjusted model (all covariates)	-0.15	-0.43 to 0.05	90%
Leave-one-out range (min to max)	-0.12 to -0.24	—	—
Excluding influential cases (Cook's D>0.18)	-0.15	-0.41 to 0.06	91%
Winsorized outliers (1st/99th percentile)	-0.17	-0.44 to 0.03	93%

All analyses examine indirect effect in Model 1 (Age → Interface Quality → Systolic BP). CrI = credible interval (Bayesian), CI = confidence interval (frequentist). P(β<0) = posterior probability that indirect effect is negative (beneficial). The primary analysis uses priors: Age→Interface N(0.15, 0.08), Interface→BP N(-1.5, 0.6), based on published meta-analyses (see Supplementary Material 2). Results demonstrate robustness: point estimates vary by <20% across specifications, and 95% intervals consistently include the primary estimate.

Supplementary Table S3. CSUQ Spanish version

CUESTIONARIO DE USABILIDAD EN SISTEMAS INFORMÁTICOS (COMPUTER SYSTEM QUESTIONNAIRE, CSUQ) ADAPTADO

Donde 1 es totalmente en desacuerdo y 7 es totalmente de acuerdo

1	En general, estoy satisfecho con lo fácil que es utilizar la app	1	2	3	4	5	6	7
2	Fue simple utilizar la app							
3	El uso de la app se volvió rapidamente intuitivo							
4	Me siento cómodo utilizando la app							
5	Fue fácil aprender a utilizar la app							
6	Creo que me volví experto rapidamente utilizando la app							
7	En caso de error, sé como continuar utilizando la app							
8	Cada vez que cometo un error utilizando la app lo puedo resolver fácil y rapidamente							
9	La información y contenidos que provee la app son claros							
10	Es fácil la experiencia de navegación de la app							
11	La información que proporciona la app pude utilizarla en mis tareas cotidianas							
12	La organización de la información de la app fue clara							
13	La interfaz de la app y el diseño es placentero							
14	Me gustó utilizar la app							
15	La app tuvo las herramientas que esperaba que tuviera							
16	En general, estoy satisfecho con la app							
